# Medical Findings in 2975 Examinations for Child Sexual Abuse: A Retrospective Review

**DOI:** 10.1111/jpc.70374

**Published:** 2026-04-07

**Authors:** Patrick Kelly, Kate Wallace, Peter Reed

**Affiliations:** ^1^ Te Puaruruhau (Child Protection Team), Starship Child Health, Health New Zealand Te Toka Tumai Auckland New Zealand; ^2^ Department of Paediatrics: Child and Youth Health Faculty of Medical & Health Sciences, Waipapa Taumata Rau, University of Auckland Auckland New Zealand; ^3^ Marinoto Child and Youth Mental Health Services, Health New Zealand Waitematā Auckland New Zealand; ^4^ Reed NZ Ltd. Rotorua New Zealand

**Keywords:** child abuse, forensic sciences, MeSH, sexual

## Abstract

**Aim:**

To describe anogenital examination findings in a large cohort seen for child sexual abuse concerns, with findings classified following current international guidelines.

**Methods:**

Retrospective review of records of the Starship Children's Hospital child protection team for all children and adolescents (0–17 years) seen over 20 years.

**Results:**

A total of 4443 children and adolescents were seen, of whom 3942 (89%) were female. The age distribution was bimodal, with a small peak in childhood and a larger peak in adolescence. Anogenital examination occurred in 2975 (67%), more often in those < 8 years (1197/1394, 86%) compared to those 8 years and older (1778/3049, 58%), *p* < 0.0001. The proportion examined declined steadily, from 93% in 1999 to 54% in 2018. 2263 examinations (76%) were normal, 425 (14%) had findings unrelated to sexual abuse and 287 (10%) had abnormal findings possibly related to sexual abuse. Of 1426 examined at Tanner Stage 1, 41 (3%) had abnormal findings, compared to 246/1549 (16%) at Tanner Stages 2–5. Of 16 pregnant adolescents examined, 2 (13%) had anogenital findings of previous trauma. In adolescents examined acutely where semen was observed on Gram stain, 8/17 (47%) had anogenital findings of acute trauma.

**Conclusions:**

The cohort seen for sexual abuse concerns has changed over 20 years, becoming increasingly adolescent. This has resulted in a decrease in the proportion examined and an increase in the rate of abnormal findings. Anogenital examinations are often normal in pregnancy or with semen present, reinforcing the international consensus that normal does not mean nothing happened.

## Summary

- What is already known on this topic
  ○ Research in the area of sexual abuse has resulted in widely accepted guidelines for interpreting anogenital appearances in children and adolescents.
  ○ When a child or adolescent seen for sexual abuse concerns is examined by expert practitioners familiar with those guidelines, the examination is usually normal.
  ○ A normal anogenital examination does not mean nothing happened.
- What this paper adds
  ○ New data on anogenital findings, described in accordance with current guidelines, in a large New Zealand cohort of children and adolescents seen for sexual abuse concerns.
  ○ New data on changing patterns of referral and examination for sexual abuse concerns.
  ○ Further data reinforcing existing evidence that normal does not mean nothing happened.

## 1 Introduction

Child and adolescent sexual abuse affects approximately one in four females and one in six males in New Zealand and Australia [1], with onset at a median age of nine to ten years [2, 3]. Many victims experience serious long‐term health effects [4, 5].

Recognition of the prevalence of child and adolescent sexual abuse first emerged during the international women's rights movement of the 1960s and 1970s [6]. By the 1980s, increasing numbers of children and adolescents were being referred for medical examination, often in the belief that anogenital examination might establish whether sexual abuse had occurred [7].

As referrals increased, paediatricians worldwide began to document anogenital anatomy with a detail never before required, and to research its relationship to sexual abuse [8, 9]. Over a period of 40 years, there has developed a largely agreed descriptive terminology for child and adolescent anogenital anatomy [10, 11]. A scientific consensus has also emerged that in children and adolescents seen for sexual abuse concerns the anogenital examination is usually normal [9, 11, 12, 13, 14, 15], although the rate of abnormality is higher when examined soon after sexual assault [9, 14, 15, 16].

Previously, we described medical examination findings in 2310 children and adolescents seen in Starship Children's Hospital, Auckland, for sexual abuse concerns from 1992 to 1998 [17]. However, since then, guidelines for classification of anogenital findings in childhood and adolescence have continued to evolve [10, 11]. The most generally accepted consensus guidelines, developed iteratively in the US, are widely known as the ‘Adams criteria’ [18].

The aim of this study was to describe the outcome of anogenital examination in a more recent cohort of children and young people seen for sexual abuse concerns, with findings described and categorised in accordance with the latest iteration of the ‘Adams criteria’ [10].

## 2 Methods

### 2.1 Setting

This study was conducted in the city of Auckland (population 1.64 million), in the multi‐disciplinary Child Protection Team (CPT) of Starship Children's Hospital. The CPT was named Te Puaruruhau by Ngāti Whātua, the Indigenous iwi (tribe) on whose whenua (land) Starship was built. The name can be translated as ‘sheltering the bud’. Since Starship opened in November 1991, Te Puaruruhau has provided 24 h a day, 7 days a week metropolitan services to children or adolescents (0–17 years) where there are sexual abuse concerns.

### 2.2 Participants

Children and adolescents come from multiple referral sources, including self‐referrals, school counsellors, school nurses, general practitioners, emergency departments, inpatient hospital services, statutory child protective services and the police. The reasons for concern vary widely—from caregiver anxiety about physical symptoms, physical findings or behavioural changes in younger children, through complex co‐parenting issues in children living between two homes to unequivocal disclosures or witnessed events. The legal age of consent in Aotearoa New Zealand is 16 years, but the law is complex [19, 20] and in practise consenting sex between peers under the age of 16 is not prosecuted by the police [21] or referred to Te Puaruruhau.

All children and adolescents seen are entered on the Te Puaruruhau database. All those seen are offered a medical examination, but we do not perform an examination if a child indicates that they do not wish to be examined, or an adolescent or the guardian of a child does not consent. Examinations are performed with a colposcope by a consultant paediatrician. Findings are documented on proforma genital diagrams for prepubertal and pubertal children and adolescents, with the hymen drawn into the diagram by the examiner, and anogenital structures and findings described and annotated systematically by hand. Routine genital photography for peer review is the standard of practise [17, 22]. Photographs are not taken if a child indicates they do not want photographs, or an adolescent or the guardian of a child does not consent. However, consent for anogenital photography for peer review is almost always able to be obtained if anogenital findings are thought to be abnormal.

Testing for sexually transmitted infections (STI) varies with age, Tanner stage and presenting concerns [23]. In adolescents, where STI are almost never of forensic significance, testing is performed with Nucleic Acid Amplification Tests, but vaginal swabs for Gram stain and culture may be taken to exclude bacterial vaginosis. If forensic samples are taken, these are collected into Forensic Examination Kits provided by the police and analysed independently by a national forensic science laboratory with appropriately sophisticated techniques.

### 2.3 Examiners

Te Puaruruhau paediatricians are all members of the Helfer Society, an international network of child abuse paediatricians [24], and currently have from 15 to 32 years' experience in child and adolescent sexual abuse examination. They are accredited through MEDSAC, a national organisation, which accredits health professionals to deliver sexual assault assessment and treatment services [25]. Te Puaruruhau paediatricians train advanced trainees and paediatricians throughout Australasia in the medical assessment of suspected sexual abuse, in courses recognised by MEDSAC and the Royal Australasian College of Physicians [26].

### 2.4 Peer Review

All examinations (including photographs) are peer reviewed weekly by all paediatricians in the team [17]. Peer review of genital photographs is blinded—that is, photographs are reviewed with no clinical history and with no comment from the paediatrician who performed the examination. A paediatrician who did not perform the examination documents the consensus opinions of all the paediatricians who did not examine the child. The case is then discussed. If differences arise in interpretation of genital photographs these are settled by consensus.

### 2.5 Clinical Records

Paper files are created, stored separately and retained indefinitely for sexual abuse concerns. Formal reports are created and archived within Te Puaruruhau.

### 2.6 Study Design

Retrospective review of records for all those seen for sexual abuse concerns from 1 January, 1999 to 31 December, 2018 identified from the Te Puaruruhau database.

### 2.7 Data Collection and Analysis

All archived reports were reviewed. If these were unavailable or unclear, the file was retrieved and reviewed. Demographic data collected included sex, age and ethnicity. Self‐reported ethnicity was prioritised following national protocols: Māori, Pacific, Asian, Middle Eastern/Latin American or African (ME/LA/A), European. If the child was examined, Tanner Stage was recorded. Anogenital findings were classified following current international consensus guidelines [10]. Data were collected on whether examinees were recorded to be pregnant, whether semen was recorded as observed on hospital laboratory Gram stain, and (if semen was observed) the number of hours between the alleged sexual assault and the examination.

Laboratory results were not reviewed, so data were not collected on how many examinees had hospital Gram stains, nor on the results of screening for STI. Analysis results from the national forensic science laboratory were not available for this study.

All data were entered into Excel. De‐identified data were imported into JMP V.15 (SAS Inc) and analysed using standard descriptive statistics.

### 2.8 Outcome Measures

Anogenital examination findings classified following current consensus guidelines [10].

### 2.9 Patient and Public Involvement

Patients and public were not involved in the design, conduct, or reporting of this study.

## 3 Results

From 1 January, 1999 to 31 December, 2018, 4443 children and adolescents were seen for sexual abuse concerns. Original reports and/or clinical records were retrieved for all 4443 patients. Of these, 2975 (67%) had an anogenital examination (Table 1).

**TABLE 1:** Anogenital examination by participant characteristics.

Characteristics of all participants			Anogenital examination performed			
	*n*	%	*n*	%	95% CI	*p*
Entire cohort						
	4443		2975	67%	66 to 68	
Cohort by decade						
1999–2008	2220	50%	1697	76%	75 to 78	< 0.0001
2009–2018	2223	50%	1278	57%	55 to 60	
Cohort by sex						
Female	3942	89%	2632	67%	65 to 68	0.48
Male	501	11%	343	68%	64 to 73	
Cohort by ethnicity						
European	1535	35%	1043	68%	66 to 70	0.1
Māori	1596	36%	1080	68%	65 to 70	
Pacific	941	21%	598	64%	60 to 67	
Asian/MeLAA^a^	371	8%	254	68%	63 to 73	
Cohort by age band (years)						
< 8	1394	31%	1197	86%	84 to 88	< 0.0001
≥ 8	3049	69%	1778	58%	57 to 60	

^a^ MeLAA stands for Middle Eastern/Latin American or African ethnicity.

### 3.1 Year of Examination, Demographics and Anogenital Examination

Relationships between demographic characteristics and whether examination occurred are shown in Table 1. The rate of examination in those seen was lower in the second decade (*p* < 0.0001). Most seen were female, and Māori were over‐represented compared to the population of metropolitan Auckland (data not shown), but no sex or ethnicity was more or less likely to be examined. Age was consistently associated with examination—younger children were more likely to be examined (*p* < 0.0001). All those < 8 years old were prepubertal (Tanner Stage 1).

### 3.2 Age Distribution

**FIGURE 1:**
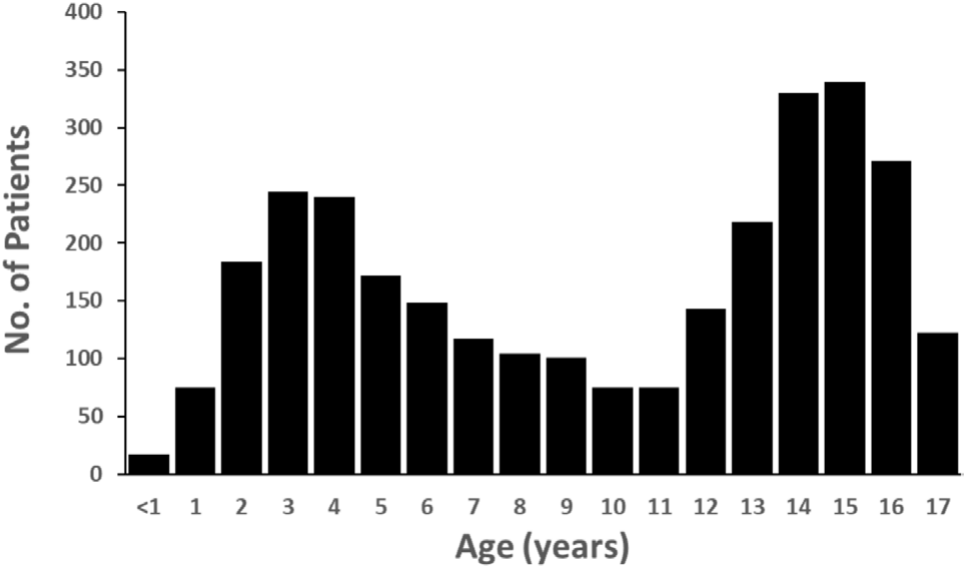
Anogenital examinations by age.

The age distribution, both in all those seen and in those examined, was bimodal, with a smaller peak in children < 8 years old and a larger peak in adolescents ≥ 12 years old. Figure 1 shows the age distribution for those examined.

### 3.3 Change in Proportion of Anogenital Examinations

**FIGURE 2:**
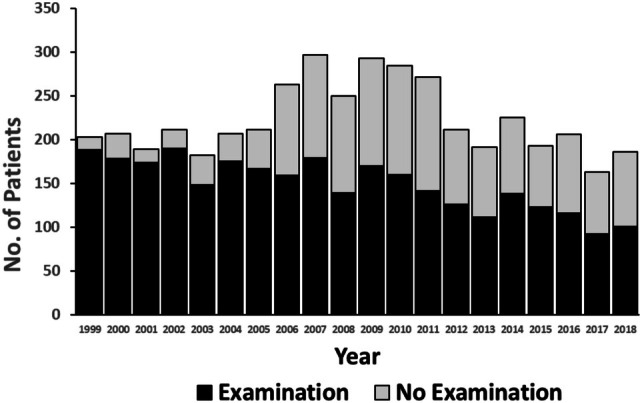
Children and adolescents seen or examined—by year.

The proportion of those seen who had an examination declined steadily over time, from 93% of those seen in 1999 to 54% of those seen in 2018 (Figure 2).

### 3.4 Anogenital Findings

**FIGURE 3:**
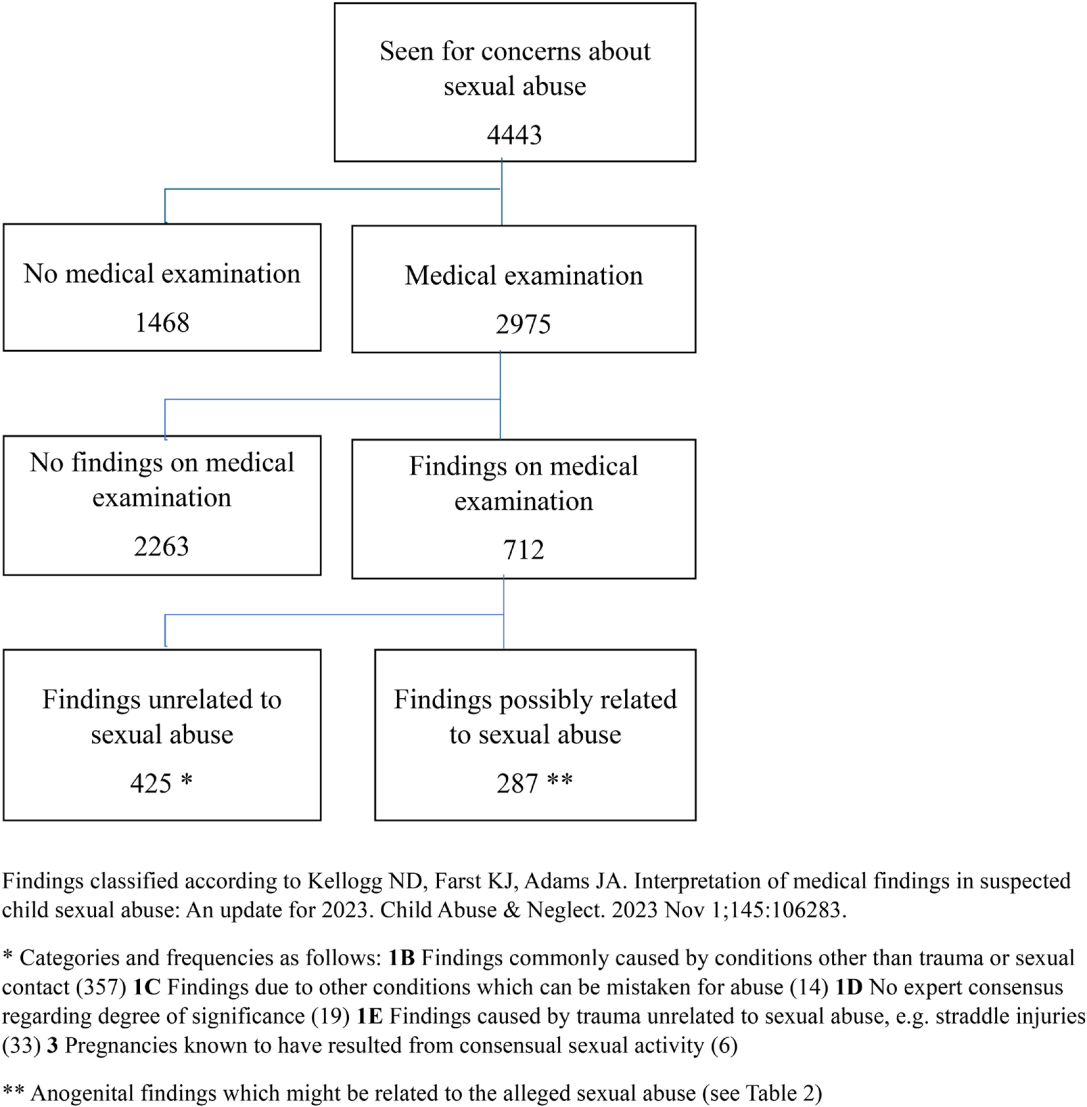
Flow diagram of anogenital examinations and findings.

Of 2975 anogenital examinations, 2263 (76%) were normal; 425 (14%) had findings unrelated to sexual abuse; and 287/2975 (10%) had findings caused by trauma or diagnostic of sexual contact possibly related to the alleged sexual abuse (Figure 3).

The frequency of each finding caused by trauma or diagnostic of sexual contact possibly related to sexual abuse is set out in Table 2. Acute injuries to the hymen, posterior fourchette, or vestibule were common. Acute perianal injuries, vaginal lacerations and perianal scars were rare.

**FIGURE 4:**
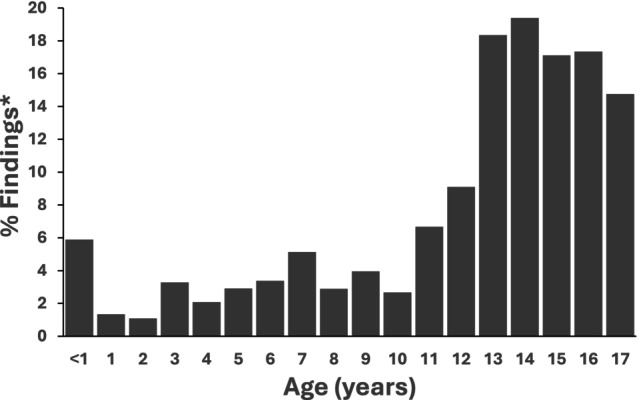
Findings of trauma or sexual contact possibly related to sexual abuse, by age. * % of those examined. Findings classified according to Kellogg ND, Farst KJ, Adams JA. Interpretation of medical findings in suspected child sexual abuse: An update for 2023. Child Abuse & Neglect. 2023 Nov 1;145:106283.

The rate of findings caused by trauma or diagnostic of sexual contact and possibly related to sexual abuse varied by age, from 1% at 1–2 years old to 15–19% at 13–17 years old (Figure 4).

**TABLE 2:** Findings of trauma or sexual contact possibly related to sexual abuse.

Finding^a^	*n* ^b^
Findings caused by trauma (Category 1E)	271
Acute laceration(s) or bruising of labia, penis, scrotum, or perineum	29
Acute laceration of the posterior fourchette/vestibule, not involving the hymen	74
Bruising, petechiae, or abrasions on the hymen	108
Acute laceration of the hymen, of any depth; partial or complete	76
Vaginal laceration	6
Perianal bruising/laceration with exposure of tissues below the dermis	8
Perianal scar	2
Healed hymenal transection/complete hymen cleft – a defect in the hymen below the 3 to 9 o'clock location that extends to or through the base of the hymen, with no hymenal tissue discernible at that location	34
Findings diagnostic of sexual contact (Category 3)	27
Pregnancy	10
Semen identified in forensic specimens taken directly from the body	17

^a^ Findings classified according to Kellogg ND, Farst KJ, Adams JA. Interpretation of medical findings in suspected child sexual abuse: An update for 2023. Child Abuse & Neglect. 2023 Nov 1;145:106283.

^b^ Individual patients may have multiple findings.

### 3.5 Pubertal Stage

There were 1426/2975 children examined at Tanner Stage 1 (48%). Of these, 41 (3%) had findings caused by trauma or diagnostic of sexual contact possibly related to sexual abuse.

There were 1549/2975 children and adolescents examined at Tanner Stages 2 to 5 (52%). Of these, 246 (16%) had findings of trauma or sexual contact possibly related to sexual abuse.

### 3.6 Pregnancy

Including pregnancies both from consensual sexual activity (6) and alleged sexual abuse (10), 16 adolescents (age 12–16 years) were pregnant at the time of examination. Of these, two (13%) had anogenital findings (healed hymenal transections) caused by trauma and possibly related to the event which resulted in pregnancy.

### 3.7 Semen on Gram Stain

There were 17 adolescents (age 13–16 years) where semen was recorded as observed on hospital laboratory Gram stain. All were seen for forensic examinations at the request of the police—15 within 24 h, and two at 42 and 52 h (median 10, inter‐quartile range 11, range 4–52 h). Eight (47%) had acute anogenital findings caused by trauma possibly related to sexual assault: three lacerations of the posterior fourchette/vestibule; four with hymenal bruising, petechiae or abrasions; three hymenal lacerations; one vaginal laceration.

## 4 Discussion

The most striking finding of this study was the change in practise with regard to anogenital examination. Whereas in the 1990s, almost all those seen for sexual abuse had anogenital examinations [17], by 2018 decisions not to examine were as common as decisions to examine. This probably reflects changing referral patterns. In this study, referrals followed a bimodal age distribution, with the larger peak in adolescence. In the 1990s, there was also a bimodal age distribution, but it was the inverse of the current pattern [17]. Then, the larger peak was in children < 8 years old. Referrals were often driven, not by a child's disclosure, but by caregiver anxiety about behaviour, physical symptoms, or the genital appearance [17]. Most children were examined, but their anogenital findings were usually variations of normal, skin conditions unrelated to abuse, or accidental injuries [17]. Although in the current study data were not collected on referral reasons, the rate of examination in prepubertal children who are seen remains high, suggesting that some caregivers still have similar concerns. However, it appears that overall, fewer prepubertal children and more adolescents are being referred.

One factor that may have influenced the changing age distribution is knowledge about a doctor's ability to verify sexual abuse. On the one hand, some caregivers may still believe the myth that sexual abuse always causes visible damage and request an anogenital examination to ‘prove’ whether sexual abuse occurred [27]. On the other hand, social workers and police may be more likely to know this is a myth. Almost 20 years ago, it was estimated that statutory child protective services in New Zealand referred children for medical assessment in fewer than 1 in 4 sexual abuse investigations [28]. Since then, increased awareness that the medical examination is usually normal may have reduced the number of prepubertal referrals even further.

In contrast, most adolescent referrals follow a disclosure by the adolescent and are more often within a forensic timeframe [15, 16]. Our experience is that many police officers—while still requesting collection of forensic samples—know that the anogenital examination will not ‘prove’ whether the event occurred or was consensual. There is no medical examination finding that will in itself determine whether an adolescent consented to sexual activity [29, 30]. For many adolescents and their caregivers, their concern is the exclusion of physical injury and prophylaxis against pregnancy and sexually transmitted infection, rather than an expectation that examination will prove that the alleged sexual assault took place. Many adolescents, especially those seen outside a forensic timeframe, when fully informed about the benefits and limitations of examination, will elect not to have an anogenital examination.

An unexpected observation was the higher rate of anogenital findings caused by trauma or diagnostic of sexual contact than in our previous study. In the 1990s the rate was 6% [17], in this study, it is 10%. However, on comparing the frequency of findings by age (Figure 4 in both papers), the frequencies are very similar. The higher overall rate in this study simply reflects the shift in age distribution towards adolescents, who are more likely to be seen within hours or days of alleged sexual assault, and thus more likely to have acute anogenital findings.

Although most studies do not report findings by Tanner Stage, our data (findings of trauma or sexual contact in 3% at Tanner 1 and 16% at Tanner 2 to 5) are not surprising. The largest studies of anogenital findings in child sexual abuse (including our previous study) all use age as a proxy for pubertal status, and in all, younger children predominate. In 2002, a study from Los Angeles reported abnormal findings in 4% of 2384 children with an average age of 6.9 years [12]. In 2016, a study from Tennessee reported abnormal findings in 7% of 1491 female children with an average age of 6.7 years. The authors reported that 72% were Tanner Stage 1 but did not describe the relationship between Tanner Stage and anogenital findings [14]. In 2018, a Canadian study reported abnormal findings in 4.8% of 3569 children overall [15]. Although the authors did not report Tanner Stage, they compared those < 12 years old (2657, 76%) with those 12 to 18 years old (823, 24%). There were abnormal findings in 2.2% of children (< 12 years), and 13.9% of adolescents (12 to 18). They also studied the relationship between abnormal findings and timing of examination and confirmed that abnormal findings were more frequent when children or adolescents were seen within 72 h of an alleged assault (14.2% versus 4.5%) [15]. For the reasons discussed above, the higher overall rate of findings in our current study most likely reflects the higher proportion of adolescents in our study.

Although the rate of abnormal findings is higher in adolescents, our data confirm that even in adolescents, the anogenital examination is usually normal. This does not mean nothing happened. In 2004, a study from Texas found that only 2/36 pregnant adolescents had anogenital examination findings diagnostic of penetration [31]. Although our rate of abnormal examination in pregnant adolescents was higher (2/16), 14/16 had no anogenital findings to ‘prove’ vaginal penetration, even though vaginal penetration is highly likely to have occurred.

The rate of anogenital findings is known to be higher when examination occurs soon after an assault [9, 15, 16]. Our study is consistent with this, in that 8/17 with semen identified had acute anogenital injuries. However, even then (a median of 10 h after an assault and with semen present), the chance of anogenital injury was only 50/50. These data further support the international consensus that a normal examination does not mean nothing happened.

### 4.1 Strengths

Strengths of this study are that it describes a large cohort from a single established centre with consistent peer‐reviewed practise, thorough documentation, and complete ascertainment (records were retrieved and reviewed for every child and adolescent seen).

### 4.2 Limitations

This was primarily a review of medical reports and further records were accessed only as required to establish Tanner Stage and anogenital findings. Laboratory results were therefore not reviewed, so data on sexually transmitted infections are not reported. Gram stain is a relatively insensitive method of detecting the presence of semen [32], and forensic laboratory data were not available, so our data on the prevalence of semen will be an underestimate.

## 5 Conclusion

The age distribution of children and adolescents seen for medical assessment in Auckland for sexual abuse concerns has changed, becoming increasingly adolescent. This may reflect increasing awareness that medical examinations in prepubertal children are unlikely to ‘prove’ whether abuse occurred. In contrast, most adolescent referrals are initiated as requests for health assessment and intervention after an adolescent has made a clear allegation of sexual assault and are not necessarily intended to ‘prove’ whether the event took place.

The rate of anogenital examination in those seen has fallen, which is likely to reflect the change in age distribution. Adolescents are more likely to choose not to have a medical examination.

The rate of abnormal findings in those examined has increased, which is also likely to reflect the changing age distribution—adolescents are more likely to be seen within hours or days of a sexual assault, and more likely to have findings of trauma or sexual contact.

However, the fact that many anogenital examinations are normal even in pregnant adolescents, and in those where semen is found, reinforces the international consensus that medical examination is often normal in children and adolescents seen for sexual abuse concerns, and that normal does not mean nothing happened [31].

## Author Contributions

**Patrick Kelly:** guarantor; conceptualisation; data curation; funding acquisition; investigation; methodology; project administration; resources; supervision; validation; visualisation; writing – original draft preparation; writing – review and editing. **Kate Wallace:** conceptualisation; data curation; investigation; methodology; resources; validation; writing – review and editing. **Peter Reed:** conceptualisation; data curation; investigation; methodology; resources; supervision; validation; visualisation; formal analysis; writing – review and editing.

## Funding

This work was supported by Starship Foundation 2021 Te Puaruruhau Research Fellowship.

## Ethics Statement

Approved by the Health and Disability Ethics Committees—Ministry of Health New Zealand, project ID 9636.

## Conflicts of Interest

The authors declare no conflicts of interest.

## Supporting information


**Data S1:** STROBE Statement—Checklist of items that should be included in reports of cohort studies.

## Data Availability

Data not available due to privacy and ethical considerations.
